# Estimation of Actual Evapotranspiration by Remote Sensing: Application in Thessaly Plain, Greece

**DOI:** 10.3390/s8063586

**Published:** 2008-06-01

**Authors:** Alexia Tsouni, Charalabos Kontoes, Demetris Koutsoyiannis, Panagiotis Elias, Nikos Mamassis

**Affiliations:** 1 Department of Water Resources, National Technical University of Athens, Heroon Polytechneiou 5, Zographou, 157 80, Athens, Greece; E-mails: alexiatsouni@yahoo.gr (A. T.); dk@itia.ntua.gr (D. K.); nikos@itia.ntua.gr (N. M.); 2 Institute for Space Applications and Remote Sensing, National Observatory of Athens, I. Metaxa & Vas. Pavlou Str., Lofos Koufou, P. Penteli, 152 36, Athens, Greece; E-mail: pelias@space.noa.gr (P. E.)

**Keywords:** Actual evapotranspiration, Remote sensing, NOAA-AVHRR images, FAO Penman-Monteith, Granger, Carlson-Buffum

## Abstract

Remote sensing can assist in improving the estimation of the geographical distribution of evapotranspiration, and consequently water demand in large cultivated areas for irrigation purposes and sustainable water resources management. In the direction of these objectives, the daily actual evapotranspiration was calculated in this study during the summer season of 2001 over the Thessaly plain in Greece, a wide irrigated area of great agricultural importance. Three different methods were adapted and applied: the remote-sensing methods by Granger (2000) and Carlson and Buffum (1989) that use satellite data in conjunction with ground meteorological measurements and an adapted FAO (Food and Agriculture Organisation) Penman-Monteith method (Allen at al. 1998), which was selected to be the reference method. The satellite data were used in conjunction with ground data collected on the three closest meteorological stations. All three methods, exploit visible channels 1 and 2 and infrared channels 4 and 5 of NOAA-AVHRR (National Oceanic and Atmospheric Administration - Advanced Very High Resolution Radiometer) sensor images to calculate albedo and NDVI (Normalised Difference Vegetation Index), as well as surface temperatures. The FAO Penman-Monteith and the Granger method have used exclusively NOAA-15 satellite images to obtain mean surface temperatures. For the Carlson-Buffum method a combination of NOAA-14 and NOAA-15 satellite images was used, since the average rate of surface temperature rise during the morning was required. The resulting estimations show that both the Carlson-Buffum and Granger methods follow in general the variations of the reference FAO Penman-Monteith method. Both methods have potential for estimating the spatial distribution of evapotranspiration, whereby the degree of the relative agreement with the reference FAO Penman-Monteith method depends on the crop growth stage. In particular, the Carlson-Buffum method performed better during the first half of the crop development stage, while the Granger method performed better during the remaining of the development stage and the entire maturing stage. The parameter that influences the estimations significantly is the wind speed whose high values result in high underestimates of evapotranspiration. Thus, it should be studied further in future.

## 1. Introduction

The increasing demand for water resources combined with stagnant supply or decreasing availability constitutes a critical problem. The need of sustainable water resources management is not questioned. In this context, the scientific research on water resources is necessary to quantify the water budget components and their spatial distribution.

Evapotranspiration is one of the main components of the water cycle and the importance of its accurate estimation is obvious, however, this is difficult to achieve in practice because actual evapotranspiration can not be measured directly and varies considerably in time and space.

A large number of more or less empirical methods have been developed over the last 50 years worldwide to estimate evapotranspiration from different climatic and meteorological variables. The analysis of the performance of the various algorithms revealed the need for formulating a standard method for the computation of the reference crop evapotranspiration. For this reason the FAO Penman-Monteith method (Allen at al., 1998) has been recommended as a standard.

The usual problem of these conventional methods is that they can only provide accurate evapotranspiration measurements for a homogeneous region around a meteorological station, and this cannot be extrapolated to other sites. However this became feasible from a technical and economical point of view by remote sensing technology. For this purpose, the estimation of actual evapotranspiration at regional scale has been widely studied in recent years by combining conventional meteorological ground measurements with remotely-sensed data. Several methods for assessing evapotranspiration have been developed at various spatial and temporal scales. These methods vary in complexity from statistical / semi-empirical direct approaches to more analytical approaches with a physical base, and finally to numerical models simulating the heat and water flux through the soil, the vegetation and the atmosphere (Kustas and Norman, 1996).

Courault et al. (2003) classify the different methods into the following categories:

– Empirical direct methods where remotely sensed data are introduced directly in semi-empirical models to estimate evapotranspiration (for example, the simplified relationship of Jackson et al. (1977), later analysed by Seguin and Itier (1983), using thermal infrared (TIR) data). It allows to characterise crop water use both at the local scale from ground measurements and at the scale of large irrigated areas from satellite data using the cumulative temperature difference (*T_s_*–*T_a_*, where *T_s_* is the land surface temperature and *T_a_* is the air temperature), also known as stress degree day.
– Residual methods of the energy budget combining empirical relationships and physical components. Most current operational models such as Sebal (Bastiaanssen et al., 1998) and S-Sebi (Courault et al., 2003) use remote sensing directly to estimate input parameters and evapotranspiration.
– Indirect methods generally using more complex models simulating the different terms of the energy budget. Remotely sensed data can occur at different levels in the input parameters to characterise the different surfaces, and assimilation procedures can be used to obtain more adequate data to compute evapotranspiration.

This study demonstrates the contribution of remote-sensing to the estimation of evapotranspiration over the irrigated plain of Thessaly, Greece. It examines a semi-empirical and a residual method and compares them to the reference adapted FAO Penman-Monteith method (Allen at al., 1998). The two methods are the Carlson and Buffum (1989) and Granger (2000) respectively, which have been adapted to integrate remotely sensed data in conjunction with surface meteorological measurements. The study focuses on the potential of the methods to be used as tools in order to estimate the irrigation needs and in particular their spatial distribution.

Conceptually, there is a difference between the Penman-Monteith method and the other two methods. The former evaluates the potential evapotranspiration whereas the latter two attempt to estimate the actual evapotranspiration. Even with this difference, the Penman-Monteith method is useful as it sets an upper limit to evapotranspiration which should not be exceeded by the other methods. In addition, the study area is a highly irrigated area where water demand is almost fully covered so that it can be assumed that, in most parts, potential and actual evapotranspiration coincide, which permits better intercomparison of methods. The systematic and intensive irrigation is an undeniable fact which has resulted to significant lowering of the aquifer level during the last years and has been widely criticized as an unsustainable practice.

## 2. Case study

**Figure 1.**
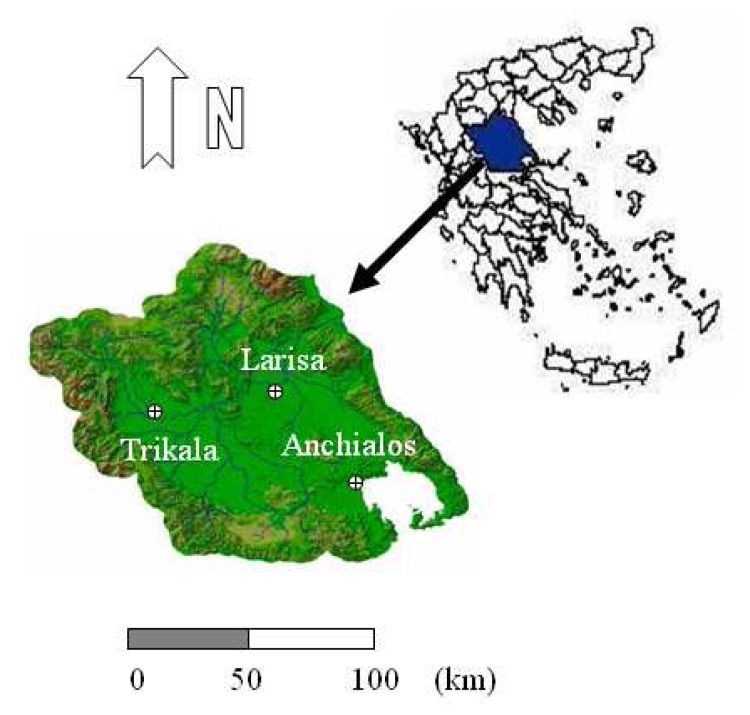
The Pinios River basin in Thessaly plain, located in Central Greece. The three meteorological stations operated by the Hellenic National Meteorological Service in Larissa, Trikala and Anchialos are illustrated.

The Thessaly plain (figure 1) was selected as the case study due to its importance for the Greek agriculture and economy. It is situated in central Greece, in the Pinios river basin, the largest river basin in Greece (area 10 700 km^2^) with mean annual rainfall of 779 mm and mean annual runoff 3500 hm^3^ (327 mm). It constitutes a region of intensive agricultural activity, where the estimation of evapotranspiration is crucial for the water resource management. The meteorological stations at some locations in the area are relatively reliable to provide the meteorological measurements needed for the calculations of the model parameters required by all three methods.

The summer season, lasting from June to August in the year 2001, was selected as the case study period, in order to estimate the irrigation needs for the plain, whose main crops are maize and cotton. The daily actual evapotranspiration was calculated for 21 days of this period uniformly distributed in the time frame of the study (7 days per month), which were selected according to a set of criteria related to the availability of satellite and meteorological data, methodological considerations and uniformity of temporal distribution.

## 3. Input data sets

### 3.1 Ground meteorological data

In the wider study area three meteorological stations are operated by the Hellenic National Meteorological Service, namely Larissa, Trikala and Anchialos.

The Larissa station is by far the most representative and useful for the assessment study since it is situated in the centre of the plain, with elevation approaching the mean elevation of the plain. On the contrary, the Trikala station is located at the western edge, in a hilly landscape, and the Anchialos station is located at the south, very close to the sea. Therefore it is justifiable to expect that the meteorological measurements in the latter two sites vary considerably in relation to the actual measurements in the interior of the plain.

For the above-mentioned reasons, the meteorological data of Trikala and Anchialos were taken into account with lower weight (half) than in the Larissa station.

### 3.2 Satellite data

The satellite data used were acquired by the National Oceanic and Atmospheric Administration (NOAA) Advanced Very High Resolution Radiometer (AVHRR) receiving stations operated by the Institute for Space Applications and Remote Sensing of the National Observatory of Athens. The value of NOAA-AVHRR sensor data for agricultural and hydrological applications has been widely recognised (Vidal and Perrier, 1989).

The FAO Penman-Monteith and Granger methods require mean daily surface temperatures, so NOAA-15 satellite images, acquired between 9:30 to 10:30 local time (UT 06:30 to 07:30) were used and the instant morning values were converted to daily ones. For the Carlson-Buffum method, NOAA-14 satellite images acquired from 7:15 to 8:30 local time (UT 04:15 to 05:30) were additionally used, since the average rate of surface temperature rise during the morning was required.

Additionally, the NOAA-15 images were used for the daily calculation of the albedo values for all the three methods. The use of pixel based spatial distribution of albedo, consists a considerable improvement over the classical approach, the latter using a single constant value per land cover type.

## 4. Assessment of evapotranspiration

### 4.1 Satellite data processing

In total, 42 satellite images were processed, covering 21 days with at least two good image acquisitions from NOAA-15 and NOAA-14 satellites. The exact dates of satellite image acquisitions are shown in table 1. Satellite data processing comprised of radiometric calibrations, geometric corrections and georeferencing to local projection system, known as Hellenic Geodetic Reference System 1987 (HGRS87, a Transverse Mercator Projection System). It also comprised image to image registrations to overcome remaining image mismatch, band reflectance normalisation to correct from sun illumination, and cloud/sea masking to keep only the valid image pixels.

All the examined methods make use of the remotely-measured albedo, the Normalised Difference Vegetation Index (NDVI) and surface temperature. The albedo was calculated as the mean value of the normalised reflectances in visible channels 1 and 2 of the NOAA-AVHRR satellite images (Vogt, 1990). The NDVI was calculated by the normalised reflectances in channels 1 and 2 (Tucker and Sellers, 1986). The following formulae were used:

(1) $$\text{ALBEDO}=\frac{R_{1}+R_{2}}{2}$$

(2) $$\text{NDVI}=\frac{R_{2}-R_{1}}{R_{2}+R_{1}}$$

The land surface temperature was estimated by the Split Window technique that uses the information conveyed in the thermal infrared channels 4 and 5 of the NOAA-AVHRR satellite images, taking into account the variability of the emission coefficients (Price, 1984, Kerr et al., 1992, Prata et al., 1995, Caselles et al., 1997). Calculations were performed only for the valid land pixels after eliminating the ones affected by clouds. For this, a cloud mask derived from the brightness temperature values of each image was applied on the data to mask out the affected pixels. The Split Window technique allows a suppression of atmospheric influences since the measurements in channels 4 and 5 are differentially influenced by the state of the atmosphere. To account also for the targets' emissivity, it was decided to make use of the algorithm introduced by Kerr et al. (1992) that uses an approximation for surface emissivity based on the NDVI. In practise the algorithm considers for each pixel a mean emissivity estimated on the assumption that this pixel is a mixture of bare soil and vegetation. Therefore the algorithm estimates the land surface temperature (*T_s_*) as follows:

(3) $$T_{s}=cT_{\text{sv}}+(1-c)T_{\text{sb}}$$

where *c* is a coefficient representing the vegetation percentage in the pixel, *T_sv_* is the temperature of a surface fully covered by vegetation and *T_sb_* is the temperature of a bare soil surface. These variables were calculated as follows:

(4) $$c=\frac{\text{NDVI}-{\text{NDVI}}_{\min}}{{\text{NDVI}}_{\max}-{\text{NDVI}}_{\min}}$$

(5) $$T_{\text{sv}}=T_{4}+2.6(T_{4}-T_{5})-2.4$$

(6) $$T_{\text{sb}}=T_{4}+2.1(T_{4}-T_{5})+3.1$$

The Kerr algorithm has been used with success over the whole of Greece in the European Commission Research project “CALamities Information System” (CALIS, Directorate General of Environment-Centre for Earth Observation - Area 3.3 of Environment and Climate Program). This project developed a system that integrates Earth Observation know-how into the process of monitoring and assessing damages in dense agricultural areas caused by climatic hazards (CALIS 2004, http://www.aurensa.es/shopwindow/intro.html). The experience of CALIS project showed that the mean uncertainty in calculating day time land surface temperatures compared to the in-situ ones was estimated to be around 2°C, due to the emmissivity influence in the calculations. The approximation was better for the night estimates, with the uncertainty being at the level of 1°C. In general the land surface temperatures were underestimated compared to the in-situ ones.

### 4.2 The FAO Penman-Monteith method

The FAO Penman-Monteith method was derived from the original Penman-Monteith equation in combination with the equations of the aerodynamic and surface resistance. It is a method with strong likelihood of correctly predicting the reference crop evapotranspiration in a wide range of locations and climates and has provision for application in data-sparse situations (Allen at al., 1998).

According to the FAO Penman-Monteith method, the crop evapotranspiration under standard conditions (*ET_c_*) is calculated by multiplying reference crop evapotranspiration (*ET_o_*) with the crop coefficient (*K_c_*):

(7) $${\text{ET}}_{c}=K_{c}{\text{ET}}_{o}$$

ETo (mm d^−1^) is calculated by the following equation:

(8) $${\text{ET}}_{o}=\frac{0.408\Delta (R_{n}-G)+\gamma \frac{900}{\text{T}+273}u_{2}(e_{s}-e_{a})}{\Delta +\gamma (1+0.34u_{2})}$$

where R_n_ is the net radiation at the crop surface (MJ m^−2^ d^−1^), G is the soil heat flux density (MJ m^−2^ d^−1^), Δ is the gradient of the vapour pressure curve (kPa °C^−1^), *γ* is the psychrometric coefficient (kPa C^−1^), *T* is the mean daily temperature (°C), *u*_2_ is the wind speed at 2 m height (m s^−1^), *e_s_*−*e_a_* is the saturation vapour pressure deficit (kPa), *e_s_* is the saturation vapour pressure (kPa) and *e_a_* is the actual vapour pressure (kPa).

In order to derive the mean daily temperature in a small area around the Larissa station, the morning NOAA-AVHRR 15 image acquisitions were used. The mean surface temperature *T*_15_, was calculated on each image using equations (2)-(6). This value was subtracted from the respective mean daily surface temperature T calculated by the conventional data of the Larissa station and the amount *dT* occurring for each day of the study period was added to each pixel of the corresponding surface temperature satellite image:

(9) $$dT=T-T_{15}$$

The crop coefficient *K_c_* was estimated on a daily basis for the entire study period according to the single crop coefficient method and a series of assumptions for the crops of the study area.

The net radiation at the crop surface *R_n_* (MJ m^−2^ d^−1^) is given by the equation:

(10) $$R_{n}=(1-a)R_{s}-R_{\text{nl}}$$

where *α* is the albedo (−), *R_s_* is the incoming solar radiation (MJ m^−2^ d^−1^) and *R_nl_* is the net outgoing longwave radiation (MJ ^−2^ d^−1^).

The parameters *G, u*_2_, *e_s_*−*e_a_*, *R_s_* and *R_nl_* were calculated according to the formulae of the method (Chapter 3, Allen at al., 1998) by the conventional data of the three meteorological stations for the 21 selected days and subsequently they were interpolated in the surface of the entire study area using a second order polynomial.

### 4.3 The Carlson and Buffum method

The Carlson and Buffum method (1989) calculates daily actual evapotranspiration *ET_d_* from the daily surface energy budget. Remotely-sensed albedo values derived from channels 1 and 2 of NOAA 15 images were used for the optimisation of the results.

This method is based on the assumption that the soil moisture and therefore the evapotranspiration is sensitive to the rate of temperature rise during the morning, that is, between 8:00 and 10:00 local time.

The corresponding equation can be written as:

(11) $$\text{ET}=R_{n}-B\prime {\left(\frac{\Delta T_{s}}{\Delta t}\right)}^{n\prime }$$

where *ET* is the daily actual evapotranspiration (cm d^−1^), *R_nd_* is the daily net radiation (cm d^−1^), Δ*T_s_*/Δ*t* is the average rate of temperature rise during the morning (°C h^−1^) and *B′*, *n′* are constants (−) depending on wind speed, surface roughness, vegetation, and reference height, estimated either by representative values or by charts.

The average rate of temperature rise during the morning Δ*T_s_*/Δ*t* was calculated dividing the difference of the surface temperature images of NOAA-AVHRR 15 and NOAA-AVHRR 14 (*T*_15_*−T*_14_) by the difference of their corresponding receiving times Δ*t*.

In order to achieve higher accuracy, the estimation of the constants *B* and *n* for vegetation (*B_v_*, *n_v_*) and bare soil (*B_s_*, *n_s_*) was based on the method's charts, not using representative indicative values, in respect to the surface roughness and the wind speed at 6.4 m height. The maximum *NDVI* value was attributed to the predominant vegetation (namely to the constants *B_v_*, *n_v_*) and the minimum *NDVI* value was attributed to the bare soil (namely to the constants *B_s_*, *n_s_*). It was estimated that *NDVI_v_* = *NDVI_max_* = 0.570 and *NDVI_s_* = *NDVI_min_* = 0.010. So, using the values *NDVI_v_*, *NDVI_s_*, *B_v_*, *n_v_*, *B_s_*, *n_s_* in the Carlson-Buffum method, the respective B and n images were calculated for each day of the study period with the application of a linear interpolation technique.

### 4.4 The Granger method

The Granger method (2000) estimates daily actual evapotranspiration applying a conventional evapotranspiration model in which some ground data are imported as well as remotely-sensed estimates of net radiation and the vapour pressure deficit using a feedback relationship with surface temperature calculated by the infrared satellite channels data.

This method is based on two assumptions: i) the feedback links between the surface and the overlying air are such that the observed surface temperature may be a sufficiently reliable indicator of the air humidity and ii) the net long-wave radiation is driven by the energy supplied to the surface, and thus, its daily values can be estimated from the incoming short-wave radiation. Therefore:

(12) $$e_{s}-e_{a}=-0.278-0.015T_{\text{ltm}}+0.668e^{o}(T_{s})$$

(13) $$R_{\text{nl}}=-4.25-0.24\;R_{s}$$

where *e_s_*−*e_a_* is the saturation vapour pressure deficit (kPa), *e_s_* is the average saturation vapour pressure (kPa), *e_a_* is the actual vapour pressure (kPa), *e_o_*(*T_s_*) is the saturation vapour pressure (kPa), *T_s_* is the mean daily surface temperature (°C), *T_ltm_* is the climatic long term air temperature in the region (°C), *R_nl_* is the net long-wave radiation (MJ m^−2^ d^−1^), and *R_s_* is the incoming short-wave radiation (MJ m^−2^ d^−1^).

Granger's equation can be written as:

(14) $$\text{ET}=\frac{\Delta \frac{R_{n}-G}{\lambda }+\gamma {\text{E}}_{\alpha }}{\Delta +\frac{\gamma }{g}}$$

where

(15) $$E_{a}=f(u)(e_{s}-e_{a})$$

(16) $$g=\frac{1}{1+0.028e^{8.045\;D}}$$

(17) $$D=\frac{E_{a}}{E_{a}+\frac{R_{n}-G}{\lambda }}$$

In the above equations *ET* is the daily actual evapotranspiration (mm d^−1^), Δ is the gradient of vapour pressure curve (kPa °C^−1^), *R_n_* is the net radiation at the crop surface (MJ m^−2^ d^−1^), *G* is the soil heat flux density (MJ m^−2^ d^−1^), *λ* is the latent heat of vaporisation (MJ kg^−1^), *γ* is the psychrometric coefficient (kPa °C^−1^), *E_a_* is the drying power of the air (mm d^−1^*), g is* the relative evaporation (−), *f* (*u*) is the wind speed function (mm d^−1^ kPa^−1^), *e_s_*−*e_a_* is the saturation vapour pressure deficit (kPa) and *D* is the relative drying power (−).

The wind speed function *f*(*u)* is calculated by the Dalton formula:

(18) $$f(u)=\frac{0.622\frac{D_{\text{wv}}}{D_{m}}\;k^{2}\;\frac{\rho_{a}}{\rho_{w}}\;u}{P\;{\left[\ln\left(\frac{z_{a}-z_{d}}{z_{o}}\right)\right]}^{2}}$$

where *D_wv_* and *D_m_* are the water vapour and momentum diffusion coefficients respectively (−), *k* is von Karman's constant (*k* = 0.4), *ρ_a_* is the air density (=1.229 kg m^−3^), *ρ_w_* is the water density (=1000 kg m^−1^), *u* is the wind speed (mm d^−1^), *P* is the atmospheric pressure (kPa), *z_a_* is the wind measurement height (m), *z_d_* is the displacement height (m) and *z_o_* is the roughness length (m), defined as:

(19) $$z_{d}=0.7\;z_{v}$$

(20) $$z_{o}=0.1\;z_{v}$$

where *z_v_* is the vegetation height (m).

Granger's method assumes that *D_wv_*/*D_m_*=1. However, this assumption is not valid as the vegetation height increases and the atmospheric stability deviates from neutrality (Lopes, 2003). For this reason, in the present application, Equation (18) is transformed into:

(21) $$f(u)=\frac{0.622}{P}\frac{\rho_{a}}{\rho_{w}}C_{\text{at}}$$

where *C_at_* is the atmospheric conductance (mm d^−1^).

Based on the values of the wind speed *u* and the vegetation height *z_v_*, the atmospheric conductance *C_at_* is estimated by Dingman's chart (Dingman, 1994) for each day of the study period. Subsequently the wind speed function can be calculated.

## 5. Results

**Figure 2.**
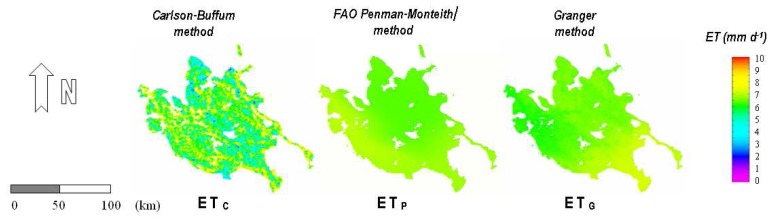
Daily actual evapotranspiration for 17/07/2001 according to the Carlson-Buffum (ET_C_), FAO Penman-Monteith (ET_P_) and Granger (ET_G_) methods

The daily evapotranspiration was calculated using the three methods for all the 21 days of the study period. As an indication, three calculated evapotranspiration images are presented for a selected date (figure 2).

**Figure 3.**
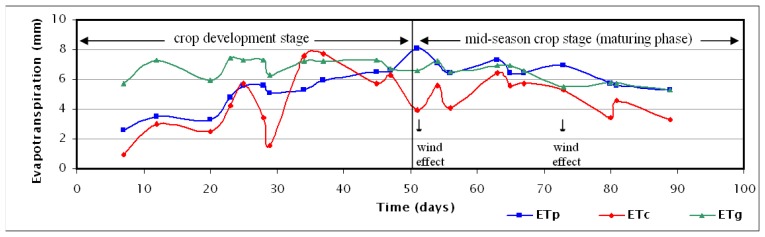
Daily actual evapotranspiration in the centre of Thessaly plain according to the three methods: FAO Penman-Monteith (ET_P_), Carlson-Buffum (ET_C_) and Granger (ET_G_) and illustration of the wind effect and the crop growth stages.

The daily evapotranspiration calculations for an area in the centre of Thessaly plain are presented both in table 1 and in figure 3 for the whole study period and for all the three methods studied. The wind speed values of the Larissa station are also shown in table 1 to emphasise the degree of influence of this parameter on evapotranspiration.

The adapted FAO Penman-Monteith method used as reference requires the conventional input data to be spatially integrated. The use of remotely sensed data enabled the areal extrapolation of the surface temperature and albedo parameters increasing the reliability of the results while making possible the estimation of the geographical distribution of the evapotranspiration assessments.

The more sophisticated Granger method as applied using the daily NOAA 15 acquisitions reproduces the general tendency the FAO Penman-Monteith method but also displays significant discrepancies at some days. During days with relatively high wind speed values the method seems to underestimate the actual evapotranspiration. On the contrary it significantly overestimates the evapotranspiration during the development stage of the crop in a systematic way. In the first half of the crop development stage the overestimation is more than 50% with a standard error rate of more than 2.5 mm. From the middle of the crop development stage to the beginning of its last fifth the error rate is reduced to 1 to 2 mm with overestimation ranging from 22 to 36%. In the last fifth of the crop development stage and in the entire mid-season maturing stage the error is maintained at less than 1.5 mm, with a deviation between −20% and +12%. If the two overestimated values due to strong wind effect are ignored (on July 21st and August 12th), the error during the end of the crop development stage and in the entire maturing stage is limited between 0 and 0.5 mm, with small deviation from −5% to +12%.

The simpler Carlson-Buffum method is also reproducing in general the tendency and the variations of the FAO Penman-Monteith method, but it yields larger deviations compared to the Granger method, which do not seem to be influenced only by the wind speed. In contrast to the Granger method, its estimates seem very satisfactory at the end of the first half of the crop development stage, where the estimation error has decreased from −1.7 mm (65% underestimation) to +0.1 mm (2% overestimation). In the second half of the crop development stage, the method shows again unsatisfactory approximation, deviating from the reference values from −3.5 to +2.3 mm (−69% to +43%). During the maturing stage, the method underestimates continuously the daily actual evapotranspiration, with an error ranging from 0.7 to 4.2 mm (−11% to −52%) from the reference estimations. If the value with the largest error 4.2 mm (due to the wind effect on July 21st) is ignored, the absolute error is reduced to levels between 0.7 to 2.3 mm (−11% to −40%), still unsatisfactory.

If the results of the methods are examined as average evapotranspiration values per crop stage (table 1), during the development stage the Carlson-Buffum method approaches the reference FAO Penman-Monteith method more closely (with 12% underestimation), while during the maturing stage the Granger method almost coincides with the reference method (with 3% underestimation). But if the results of the methods are considered for the total period, larger deviations are taken for both methods (19% and 16%), so it is preferable to distinguish the efficiency of the methods per stage or even per parts of a stage.

In certain cases large deviations from the daily actual evapotranspiration were observed near the boundaries of the study area.

## 6. Conclusions and discussion

The combination of ground and remotely sensed data is extremely important in areas with insufficient in-situ networks for monitoring the meteorological variables as well as actual evapotranspiration. The satellite data provide spatially distributed estimates of albedo, normalised difference vegetation index and surface temperature, enabling the water managers to estimate the water evaporating per day. The accuracy of the methods estimating the regional evapotranspiration is expected to increase even more if data from different satellite sensors with better radiometric and spatial resolution are integrated and combined with the NOAA-AVHRR imagery, such as the LANDSAT Thematic Mapper (TM), SPOT HRV or ASTER sensor data for deriving detailed land cover maps in order to obtain accurate estimates of the spatial distribution of the crop relating parameter.

In this study, the daily evapotranspiration was calculated during the summer season of 2001 over the irrigated plain of Thessaly, Greece. Three different methods were adapted and applied: the remote-sensing methods by Granger (2000) and Carlson and Buffum (1989) that use satellite data in conjunction with ground meteorological measurements and an adapted FAO Penman-Monteith method (Allen at al., 1998), which was selected to be the reference method. The satellite data were used in conjunction with ground data collected on the three closest meteorological stations.

The reference FAO Penman-Monteith method was adapted by spatially integrating the conventional input data, such as the surface temperature and the albedo, using their remotely sensed values. The surface extrapolation of these variables enabled the estimation of the geographical distribution of the evapotranspiration assessments and increased the reliability of the results.

However, the other two methods that rely more on remote sensing did not result in overall reliable estimates. The Granger method has a stronger theoretical background and was more stable in comparison with the Carlson-Buffum method. However, the Granger method proved to be not reliable in the first crop growth stage, in which it gave actual evaporation rates greater than the reference FAO Penman-Monteith that is considered as the ceiling (potential evapotranspiration). On the other hand, the Carlson-Buffum method is simpler and requires fewer conventional input data; nevertheless it requires two satellite images per day for deriving daily temperature estimates. As a general conclusion it is preferable to distinguish the efficiency of the methods per stage or even per parts of a crop growth stage. The Carlson-Buffum method performed better in assessing the daily actual evapotranspiration during the first half of the crop development stage, while the Granger method performed better during the remaining of the development stage and the entire maturing stage.

Consequently, since there is no obvious best method, it is recommended that both methods are further examined and developed. Other methods could also be proposed and applied in order to check their response overall or at the different crop growth stages. In any case, the results should be cross-validated with ground measurements and the reference FAO Penman-Monteith method should always be considered as the ceiling. The role of wind speed should also be examined thoroughly, since it is the factor that has the biggest influence on the reliability of the methods with remote sensing input. In brief, there is undoubtedly big room for further research and improvements.

## Figures and Tables

**Table 1.** Daily actual evapotranspiration in the centre of Thessaly plain according to the three methods: Carlson-Buffum (ET_C_), FAO Penman-Monteith (ET_P_) and Granger (ET_G_), the corresponding deviations from the reference method, as well as the average values per crop growth stage and in total. Wind speed from Larissa station is also presented.

**Crop Growth Stages**	**No**	**Date**	**Daily actual evapotranspiration *ΕΤ*for area in the centre of Thessaly plain**					**Wind speed from Larissa station*u*_2_(m s^−1^)**
			**Carlson-Buffum method *ET****_C_*		**FAO Penman-Monteith method*ET****_P_*	**Granger method *ET****_G_*		
			Actual evapotranspiration (mm/day)	Deviation from reference	Actual evapotranspiration (mm/day)	Actual evapotranspiration (mm/day)	Deviation from reference	
Crop Develolpment	1	07/06/2001	0,9	−65%	2,6	5,7	+119%	1,54
	2	12/06/2001	3,0	−14%	3,5	7,3	+109%	0,58
	3	20/06/2001	2,5	−24%	3,3	5,9	+79%	0,96
	4	23/06/2001	4,2	−13%	4,8	7,4	+54%	1,15
	5	25/06/2001	5,7	+2%	5,6	7,3	+30%	2,21
	6	28/06/2001	3,4	−39%	5,6	7,3	+30%	1,73
	7	29/06/2001	1,6	−69%	5,1	6,3	+24%	1,88
	8	04/07/2001	7,6	+43%	5,3	7,2	+36%	1,64
	9	07/07/2001	7,7	+31%	5,9	7,2	+22%	1,78
	10	15/07/2001	5,7	−12%	6,5	7,3	+12%	0,96
	11	17/07/2001	6,3	−5%	6,6	6,7	+2%	1,44
	Stage average		4,4	−12%	5,0	6,9	+38%	
Mid-Season (Maturing Phase)	12	21/07/2001	3,9	−52%	8,1	6,6	–19%	**2,65**
	13	24/07/2001	5,6	−21%	7,1	7,2	+1%	1,68
	14	26/07/2001	4,1	−36%	6,4	6,5	+2%	1,30
	15	02/08/2001	6,4	−12%	7,3	6,9	–5%	1,78
	16	04/08/2001	5,6	−13%	6,4	6,9	+8%	1,06
	17	06/08/2001	5,7	−11%	6,4	6,6	+3%	1,06
	18	12/08/2001	5,3	−23%	6,9	5,5	−20%	**3,08**
	19	19/08/2001	3,4	−40%	5,7	5,8	+2%	1,25
	20	20/08/2001	4,6	−18%	5,6	5,7	+2%	0,87
	21	28/08/2001	3,3	−38%	5,3	5,3	0%	1,35
	Stage average		4,8	−26%	6,5	6,3	−3%	

	Total average		4,6	−19%	5,7	6,6	+16%	
